# Understanding Quantitative Circadian Regulations Are Crucial Towards Advancing Chronotherapy

**DOI:** 10.3390/cells8080883

**Published:** 2019-08-13

**Authors:** Debajyoti Chowdhury, Chao Wang, Ai-Ping Lu, Hai-Long Zhu

**Affiliations:** 1 HKBU Institute for Research and Continuing Education, Shenzhen 518057, China; 2 Institute of Integrated Bioinfomedicine and Translational Science, School of Chinese Medicine, Hong Kong Baptist University, Hong Kong 999077, China

**Keywords:** circadian rhythms, transcriptional dynamics, molecular clockwork, chronotherapy

## Abstract

Circadian rhythms have a deep impact on most aspects of physiology. In most organisms, especially mammals, the biological rhythms are maintained by the indigenous circadian clockwork around geophysical time (~24-h). These rhythms originate inside cells. Several core components are interconnected through transcriptional/translational feedback loops to generate molecular oscillations. They are tightly controlled over time. Also, they exert temporal controls over many fundamental physiological activities. This helps in coordinating the body’s internal time with the external environments. The mammalian circadian clockwork is composed of a hierarchy of oscillators, which play roles at molecular, cellular, and higher levels. The master oscillation has been found to be developed at the hypothalamic suprachiasmatic nucleus in the brain. It acts as the core pacemaker and drives the transmission of the oscillation signals. These signals are distributed across different peripheral tissues through humoral and neural connections. The synchronization among the master oscillator and tissue-specific oscillators offer overall temporal stability to mammals. Recent technological advancements help us to study the circadian rhythms at dynamic scale and systems level. Here, we outline the current understanding of circadian clockwork in terms of molecular mechanisms and interdisciplinary concepts. We have also focused on the importance of the integrative approach to decode several crucial intricacies. This review indicates the emergence of such a comprehensive approach. It will essentially accelerate the circadian research with more innovative strategies, such as developing evidence-based chronotherapeutics to restore de-synchronized circadian rhythms.

## 1. Introduction

Adapting to the environment across different geophysical locations is a spontaneous tendency of life on the Earth prompted by progressive evolution. Axial rotation of Earth causes day/night cycles around 24-h. This brings periodic fluctuations in light exposure, light intensity, and environmental temperature every day. It influences all organisms immensely in terms of many aspects of their physiology and behavior. To adopt such periodical changes, most organisms have been equipped with internal biological clocks that antedates day and night cycles. It facilitates them to optimize their intrinsic physiological activities and behavior. This inherent phenomenon is termed circadian rhythm. It consists of a self-sustained 24-h rhythm generator, orchestrating the internal oscillators to the external stimuli. Recent studies have suggested that circadian systems in mammals consist of several internal clocks across the entire body [1]. These clocks, located in different tissues, are connected to the central clock, known as the suprachiasmatic nucleus (SCN). This systematic network of clocks helps in balancing various physiological processes in the body.

In this review, we have sketched the fundamentals of circadian rhythms, the molecular mechanisms of the clock, and recent advancements. We have also focused on the recent advancements in temporal synchronizations among the master clock and the peripheral clocks over different tissues. Currently, the basis of such synchronizations is not very well elucidated and there are several gaps yet to be addressed. Therefore, we reviewed the panoramic standpoints of the circadian research to consolidate our understanding from molecular level to systems level. In future, such deep understanding would certainly help us in reimagining the potential scopes of chronotherapeutic interventions.

## 2. Molecular Insights of the Clockwork and the Feedback

Most organisms on Earth exhibit the inherent spontaneous feature to adapt to environmental entrainments differentially. Remarkably, to achieve such adaptability, most of the organisms possess an internal biological clock that predates day and night cycles and facilitates in balancing physiological activities and behavior. This inherent physiological system is known as circadian rhythm [2]. The 24-h circadian systems synchronize internal oscillators to external stimuli such as light and temperature, also known as the zeitgebers or timekeepers. This drives temporal orchestrations of multiple physiological processes [2].

### 2.1. The Overview of the Mammalian Circadian Clockwork and Its Regulations

**Figure 1:**
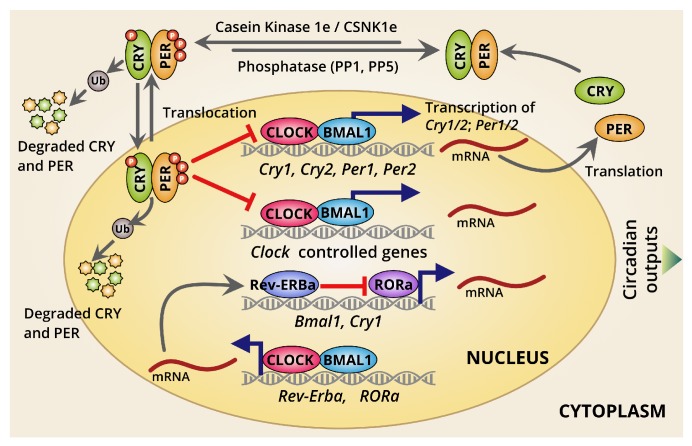
A typical circadian clockwork involved in mammalian cells. It consists of two clockworks, the primary and the secondary TTFLs. A set of core clock genes in those two TTFLs are essential for producing the 24-h self-sustained indigenous oscillations. The primary TTFL composed of CLOCK, BMAL1, CRY, and PER. The CLOCK:BMAL1 heterodimer induces the clock-controlled genes and CRY and PER act as negative regulators for their own transcriptions. In the secondary TTFL, the *Rev-Erbα* and *RORα* are induced by the CLOCK:BMAL1. Their product ROR*α* counteracts with the primary clock genes.

Recently, core molecular mechanisms beyond the rhythmic oscillations have been well defined. This is primarily generated by two interconnected transcription/translation feedback loops (TTFLs) [2,3,4]. These loops operate together to produce robust 24-h rhythmic gene expression patterns. These canonical molecular clockworks include a core set of genes being negatively regulated through the TTFLs (Figure 1). In addition, there have been two interconnected TTFLs that have been recognized in mammalian circadian systems—the primary and the secondary TTFLs. The temporal control of the core primary TTFLs was majorly intended by four integral clock-related proteins: two activators (CLOCK and BMAL1) and two repressors (PER and CRY), along with kinases and phosphatases as rate-limiting factors to sustain the closed-loop feedback regulation. The molecular interconnection among those regulators are essential for the circadian clockworks.

### 2.2. Dynamics of Circadian Transcription/Translation Feedback Loops

Understanding the dynamics of the circadian transcription must provide a robust perception of the clockworks at molecular level. The two major transcriptional activators, CLOCK and BMAL1, form a heterodimeric complex (CLOCK:BMAL1) and are an essential positive regulator for mammalian circadian clockworks. This heterodimer is capable of binding to several thousand sites across the genome in a timely manner [4,5]. Recruitment of integral clock protein transcriptional repressors, PER1, PER2, CRY1, and CRY2 is facilitated upon CLOCK:BMAL1 heterodimeric complex binds to the consensus E-box DNA motifs [2,4]. This process usually occurs during the dark phase [2,4]. The most recent model suggested that cyclical repression of CLOCK:BMAL1 activity by another heterodimeric complex PER:CRY confers the rhythmicity of transcriptional output over ~24-h periodicity, which were further extended beyond CLOCK:BMAL1 target genes through circadian regulation of additional transcription factors and coregulators [6]. Also, PER and CRY proteins used to get recruited to other different sites across the genome which are usually enriched for nuclear hormone receptor-binding [6,7]. This gives PER and CRY control over the transcriptional activities of a diverse array of nuclear hormone receptors [6,7]. Thus, PER and CRY strongly demonstrate an extensive temporal regulation mediated by different TFs outside the core molecular clock loop. This has also indicated the most plausible mechanism of conveying the temporal cues to the downstream TFs through a rhythmic transcriptional regulation by clock-driven oscillations over the 24-h in a day. PER is used to regulate or limit circadian transcriptional machinery in two different modes: (1) PER facilitates a direct interaction with the CLOCK:BMAL1 to recruit some other components, including epigenetic modulators such as histone deacetylases to repress further transcriptional activation by CLOCK:BMAL1 [8]. (2) By interacting with RNA-binding proteins and helicases, PER gets recruited to the termination site of the *Per1*, directing transcriptional termination to limit CLOCK:BMAL1-mediated transcriptions independently [8].

A few studies have also suggested CRY as a key player in potential repression of CLOCK:BMAL1 driving the transcriptional activation [2,4]. Researchers have identified two cryptochromes, CRY1 and CRY2, in mammals. Out of those two, CRY1 has been widely studied. CRY1 alone has been found to sustain circadian rhythms and exhibit a significantly altered expression relative to CRY2. CRY2 is usually encoded by multiple clock-regulated promoter elements [9,10]. In fact, CRY1 is used to interact with CLOCK:BMAL1 heterodimer autonomously ahead of PER, and is usually found at CLOCK:BMAL1-bound sites in the early morning. Thus, CRY1-mediated transcriptional control to maintain CLOCK:BMAL1-mediated transcriptional activation is used to react as a premier molecular gatekeeper, and is presumed to be more robust, self-sustained, and independent by features. However, a thorough mechanism is not yet explained enough.

In addition to this primary core TTFL, another secondary TTFL clockwork has been recognized. This is generated through transcriptional activation by the retinoid-related orphan receptors (ROR*α*, ROR*β*, and ROR*γ*) [2,11] and repression by REV-ERB*α*/REV-ERB*β* [12,13]. This second loop drives rhythmic changes in *Bmal1* transcription and introduces a delay in *Cry1* mRNA expression that counterbalances it from the CLOCK:BMAL1 heterodimer-regulated genes. These rhythmic changes in BMAL1 abundance are not primarily required to drive the core TTFL [2]. However, to maintain the circadian timing and timely ticking of the internal clocks, the second TTFL, the ROR/REV TTFL-induced delay in *Cry1* expression is very crucial [12,13]. It provides an additional layer of fine-tuning of the body clocks to be maintained in a timely manner keeping pace with external cues and internal physiology. Thus, interlocking feedback loops provide robustness against noise and environmental perturbations to maintain accuracy in circadian timing. The existence of these cooperatives also helps to generate phase delays in circadian transcriptional output, which temporally regulates the expression of clock-controlled output genes and other circadian rhythm-related genes for tuning internal physiology [2]. Furthermore, there may exist several other modes of transcriptional controls beyond those two primary and secondary clockworks.

### 2.3. Different Modes of Circadian Transcriptional Regulation

To provide a more holistic overview about the circadian oscillation generation, interestingly, computational biologists and bioinformaticians have identified a few regulatory motifs associated with the clock-specific TFs to explain the circadian transcriptions [5,14]. Recently, a quantitative viewpoint with the TF-binding occupancy has refreshed the understanding on circadian gene regulations. This influences the degree of rhythmic oscillations over the day [14]. Therefore, the research track on the spatio-temporal regulations of the circadian genes may be accelerated through synergistic remodeling of circadian epigenetic landscapes. Besides, several genome-wide studies revealed an interesting fact that both the chromatin remodeling and RNAP-II recruitment are capable of demonstrating a daily rhythmic discrepancy at all actively expressed circadian genes, even if some genes do not undergo vigorous transcriptional variations [15,16]. Thus, a robust variation in epigenetic circumstances could fund an overall intensification in transcriptions over a specific period within the 24-h day. Thus, the dynamic behaviors of circadian gene expression are highly associated with the tuning of indigenous physiological clock/timing provided by the body’s own clock. Therefore, the transcriptional dynamics and variation in transcriptional kinetics over the 24-h a day used to be believed to exert a substantial role in the emergence of rhythmic properties in the mammalian clock [15,16].

However, a profound direction backing these challenges is that *cis*-acting regulatory elements play a pivotal role in restructuring the kinetics of transcriptional bursts, contributing to the circadian transcriptional variability, and/or noise [15,16]. This could be achieved through recruitment and retention of general transcriptional machinery and coregulators that facilitate restructuring the local molecular environment. Nevertheless, comprehensively decoding the detailed transcriptional snapshots and other layers of molecular regulations among the core clock and peripheral clocks remained out of reach of prevailing approaches [2]. To date, there is neither any strong scientific evidence nor any solid, comprehensive recommendation on this. The suggested tracks are limited by the complexities of more integrative algorithm developments to accommodate different layers of molecular information into a single framework. Also, only the transcriptional mode of control is not enough, and scientists must envisage more molecular complexity to understand full inclusively about circadian rhythms at the dynamic scale. Therefore, probing post-transcriptional and post-translational modifications are also quite important in addition to the transcriptional landscape.

## 3. Post-Transcriptional and Post-Translational Controls over the Molecular Oscillations

Post-transcriptional and post-translational modifications have been believed to a very important decisive determinant for regulating molecular clockwork/oscillation. Studies have suggested that approximately 80% of mRNA did not exhibit circadian rhythm in their de novo transcription; however, they do demonstrate circadian rhythms in their content [4]. This phenomenon states that post-transcriptional regulation certainly plays a critical role in executing circadian rhythmic features [17]. Another interesting insight about the emergence of post-translational modifications has been explained well in terms of affecting period length. The first mammalian clock gene, *Tau*, was identified in a mutant hamster exhibiting shortening circadian period [18]. This *Tau* possesses a missense mutation at the phosphorylation site of CK1*ϵ*, which also facilitates phosphorylation of many clock proteins, including the CRY and PER [19]. Interestingly, the patients with advanced-sleep-phase syndrome, with shorter circadian period length, have been screened to possess a missense mutation at the CK1*ϵ* site in their *Per2* gene [20]. Later, studies supported the hypothesis that the shorter period for mutated *Tau* was due to gain-of-function mutation that induced hyperphosphorylation in different sites of the PER2, resulting in shorter molecular oscillation, thus shorter period length [21]. It is not only CK1*ϵ*; there are several other kinases that can account for affecting period length [22]. Also, different kinds of post-translational modifications are imparted into circadian period maintenance, such as ubiquitination [23,24], acetylation [25], and SUMOylation [26].

Additionally, many mutations have been identified against different types of sleep/wake rhythmic disorders [27,28,29]. In recent years, research focus has gained attention towards targeting clock genes underlying mood disorder [30,31,32] and also neurodevelopmental disorders [33,34,35] for therapeutic interventions. Moreover, multiplexed molecular interactions, feedback loops, and signaling systems together offer the stability towards circadian homeostasis. However, intense study is required to comprehensively decode multi-layered mechanisms maintaining the clockwork from the molecules to cells to systems [36].

## 4. Light Entrainment and Synchronization of Biological Clocks

**Figure 2:**
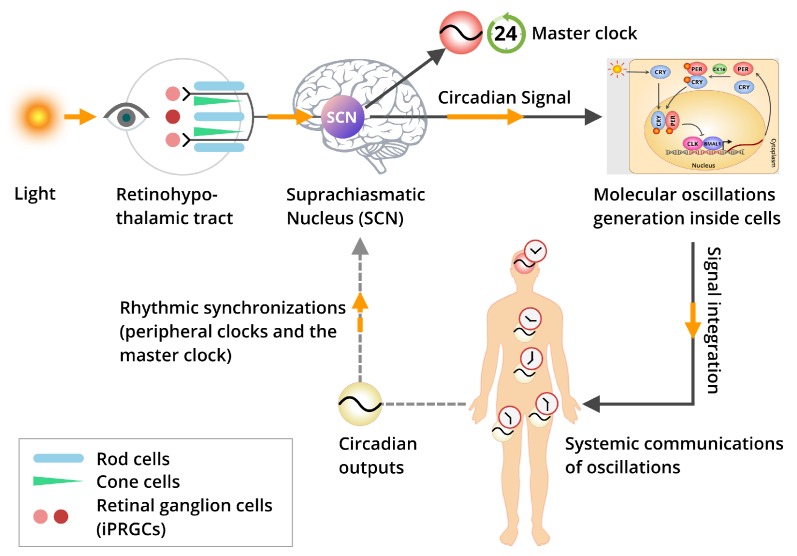
Schematic representation of the light entrainment and intercellular signal transmissions. The mammalian circadian systems are most sensitive to the light and dark cycles around the 24-h a day. The light serves as the most inevitable external zeitgeber to entrain the mammalian circadian rhythms. Usually, the photic signals from the light sources are received by the ipRGCs and reach the master clock, located at the SCN under the hypothalamus of the brain *via* RHT. In SCN, the primary oscillations are produced and get transmitted to the cellular-molecular level of distal peripheral tissues across the organism’s body. Thus, the different tissue-specific circadian outputs are produced. In addition, the locally regulated peripheral rhythms are integrated together to maintain circadian homeostasis, keeping a synchronization to the master clock.

How the light or any other form of entrainments pass from environment and get received and assimilated by physiological systems is quite interesting to know, as is the entrainment influence to be further passed onto cellular and then molecular levels to generate the self-sustained oscillation. In turn, that oscillation from molecules is synchronized again to systems. Our body’s rhythmic program and indigenous circadian clock are primarily generated in the SCN, the master clock and primary timekeeper, and then synchronized with the different peripheral clocks at distal tissues (Figure 2). The tuning between the body’s internal clocks and the external entrainments are not clear yet. A wide range of studies have postulated that the retina, especially the “intrinsically photosensitive retinal ganglion cells” (ipRGCs) act as a receiver of photic signals as input and transmit these signals to the SCN to produce the first tick in the master clock [37,38,39]. Sequentially, the SCN is used to harmonize its own cellular clocks and synchronize different peripheral clocks at distal tissues to regulate their diurnal rhythms [37,38,39]. The signals from SCN has been presumed to communicate through humoral factors and the peripheral autonomic nervous system to influence the ticking of peripheral clocks [40,41,42,43]. Finally, the molecular oscillations orchestrate the rhythms from molecule to cell, and then cell to systems. Thus, the physiological circadian homeostasis is believed to be balanced from geophysical time and external entrainments to tuning the indigenous rhythms. Eventually, studies suggested that the clock-controlled circadian genes’ regulations are quite imperative in this aspect.

However, the entire program of circadian gene expression is quite extensive throughout the body and most peripheral tissues can generate apparently independent circadian oscillations [44]. This has raised a question about the efficiency of synchronization features of circadian rhythms [41]. Peripheral clocks are used to become efficiently influenced and synchronized both by the SCN and by external environmental cues, such as light, temperature, feeding habits, and physical activities. On the other hand, the peripheral clocks are used to exert a substantial influence in controlling relevant physiological outputs: metabolism, hormonal regulation, and storage functions, and so on [45]. In return, the cumulative effect of these tissue-specific timekeeping functionality constructs an ultimate feedback to the SCN. Therefore, the circadian programs at systems levels of an organism has been considered to be a network of interrelated oscillators and feedback loops. Nevertheless, the relationship between master clock and peripheral clocks as well as relationship among different tissue-specific peripheral clocks are still ambiguous [46]. Hence, the mechanism by which they all are being timely synchronized is an active area to be investigated further. Also, the comprehensive knowledge about tissue-specific circadian regulations and harmonization of those clocks need sincere attention to maximize the use of our body’s own clock. This can lead to attaining optimum physiological output and to enrich the field of chronotherapy to treat and/or reverse the numerous circadian rhythm disruption-associated disorders such as sleep disorders, mood disorders, depressions, anxieties, seasonal-affective disorders, and jet lag. Cumulatively, this arena demands the emergence of disruptive ideas to be deployed for directive advancement in real-time practice of medicine.

As mammalian circadian systems are truly complicated, it can be considered to be composed of numerous clocks as there are cells in the entire organism. Interestingly, a prominent question arises, specifically how all these clocks (virtually representing different time zones in different tissues) get synchronized to each other and to the SCN-based master clock. The SCN is a tiny region, located underneath the hypothalamus, and above the optic chiasm of the brain. It consists of a network of functionally and phenotypically differentiated cells [47]. This SCN network functions as a master pacemaker for controlling the body’s indigenous rhythms, also known as circadian rhythms, being at the top of the structural hierarchy of the circadian systems (Figure 2) [48]. Also, this region is important for rhythmic hormone secretion and locomotor activity [49].

The SCN-based master clock facilitates orchestrating the other distal tissue-specific peripheral clocks, and thus facilitates entrainments of different signals from external cues as well as internal cues. Among the external cues, the environmental light/dark cycle has a large impact in modulating the circadian systems and synchronizations of physiological parameters. Usually, the light or photic signals are received through a special type of retina-based neuronal cell, ipRGC. They stimulate the secretion of the photopigment, melanopsin. These ipRGCs transmit the photic signal to SCN directly using RHT (Figure 2). These monosynaptic RHT fibers end in the ventrolateral region of the SCN, where the neurons primarily express vasoactive intestinal polypeptide. Studies suggest that light stimulation of the retina during the night leads to the release of glutamate, an excitatory neurotransmitter and a neuropeptide, pituitary adenylate cyclase-activating protein at the synaptic terminal of the RHT, prolonging the signal transmissions up to the SCN (Figure 2) [50].

This systematic path of signal transduction activates several molecular signaling pathways that induce chromatin remodeling and influence the molecular clockwork [51]. This is how the external cues exert an impact on molecular clockwork and its component genes to influence the circadian clock-mediated phase modulations [52]. The individual cellular oscillations are integrated together to produce a persistent circadian oscillation within the SCN [52]. Also, other brain regions, such as hypothalamus, amygdala, hippocampus, habenula, and olfactory bulbs, have been reported to exhibit daily rhythms [53]. In fact, the tissues, with neuroendocrine functions, such as arcuate nucleus, the paraventricular nucleus, and the pituitary gland, have been found to produce robust circadian rhythmic effects. These different non-SCN brain regions have been suggested in assisting circadian homeostasis maintenance through neuronal circuits [54]. These circuits are critical for keeping circadian oscillations essential as well as for regulating different crucial physiological activities over the 24-h time period, including but not limited to the integration of feeding information, redox sensing, metabolisms, and reward-driven behaviors occurring on a daily basis [55]. Thus, the understanding of the circadian systems in terms of coupling among the master core pacemaker and the peripheral clocks is quite essential. It is also crucial to elucidate the multifaced network effects on different physiological activities.

## 5. The Inter-Relation Among the Core Circadian Pacemaker and the Peripheral Clocks

The circadian system is hierarchically organized with the SCN network-based master pacemaker in the central nervous systems. This is sequentially entrained to the light every day around the 24-h and engages in conducting a distributed network of peripheral clocks across different distal cells and tissues in the body. The master clock is vital for maintenance of the sleep/wake cycle related to dark/light exposure and also essentially maintains many other physiological activities, including learning, rewarding, and neurogenesis. Again, the peripheral clocks are entrained to the master clock. However, during aging, shift work, jet lag, and in any diseased conditions, the peripheral clocks and the master clock become de-synchronized [56,57,58,59,60,61,62]. Systematic disruptions of circadian rhythms are associated with impairment of sleep behaviors, and also molecular pathogenesis of different metabolic syndromes, obesity, diabetes, and even cancer.

**Figure 3:**
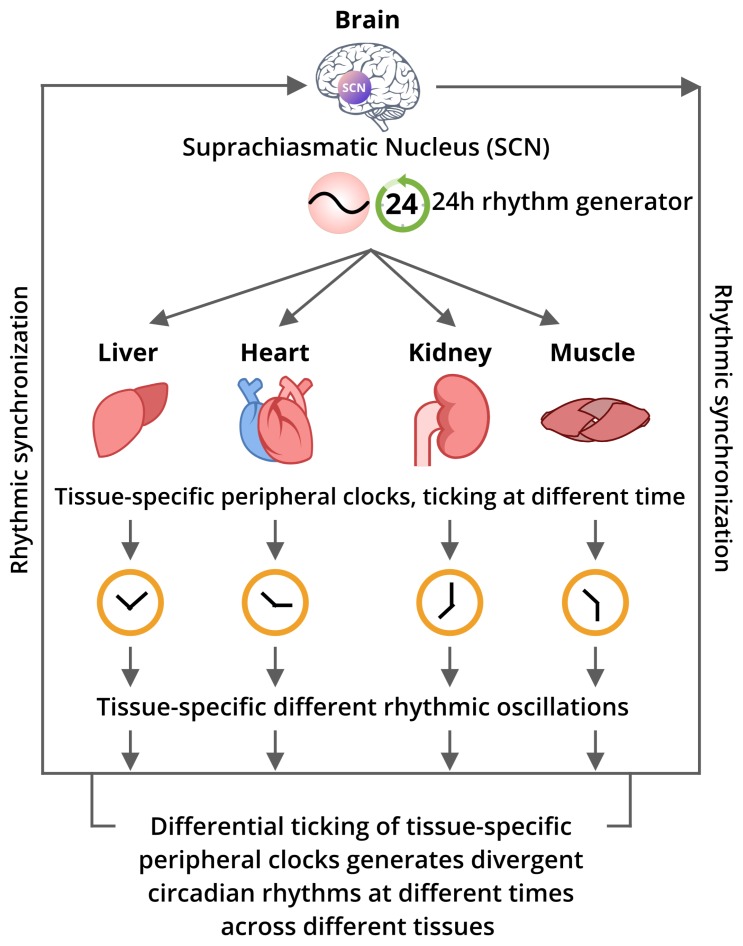
A representative connectome of the master clock and tissue-specific peripheral clocks. The primary rhythmic oscillation that is usually produced at the master clock is transduced to different tissues through the humoral and neural connectivities. The peripheral clocks preserve their own local timing and reproduce differential rhythmic oscillations, and thus differential circadian outputs. There may exist a tricky rhythmic synchronization mechanism among those peripheral clocks with the master pacemaker to establish the circadian homeostasis.

An interesting perspective on how the multiple peripheral clocks located at distal tissues exert robust controls to their circadian genes’ expressions at dynamic scales is challenging to be elucidated (Figure 3). However, several microarray experiments suggested wide ranges of transcriptional controls regulating peripheral clocks, coordinating with tissue-specific functions and temporal control [63,64]. Furthermore, many experiments confirmed that circadian output is primarily controlled at the transcriptional level [65]. However, it is quite important to know how these only few components belonging to same molecular architecture are capable of such divergent gene expressions in a tissue-specific manner. Also, the mechanisms beyond their intricated temporal tuning must be enlightened further, as the transcription-driven model alone could not completely explain the divergency of circadian rhythms [2,14,66]. Thus, the smart approach demands more layers of information to be involved in a single model to investigate the complex rhythmic oscillation. Moreover, it has been believed that post-transcriptional, translational, and/or post-translational regulation must have very significant influence towards regulating the circadian output. Recently, many research groups have been engaged in genome-wide studies to examine the temporal recruitment of integral clock proteins, transcriptional machinery, and epigenetic modifications to chromatin structure to determine how the clock confers temporal control over transcriptional output [4,10,67,68]. In fact, a few studies stated that most of the circadian changes in mRNA levels may result from post-transcriptional regulation [4,5,10,14,67,68].

However, the all-encompassing mechanistic insights underpinning such divergent circadian gene regulations (transcriptionally and/or post-transcriptionally) are not yet clearly exposed. Simultaneously, another intriguing fact is still unknown, which is how these various levels of regulations and their molecular regulators are synchronized among different tissues to exert circadian controls over physiological activities. It is also important to decode the mechanisms that lead to circadian disruption-related disorders. Hence, a research approach, integrating various high-throughput biological data, may gather deep information from the molecular aspects [69]. Moreover, such advancements must augment knowledge linked to circadian biology and physiological outcomes, especially the sleep physiology.

## 6. Circadian Biology and Human Health

Day-to-day physiological activities, such as regulating sleep patterns, feeding behavior, hormone release, hormonal homeostasis, blood pressure, and body temperature maintenance are greatly influenced by circadian rhythms. Among them, circadian rhythm-associated sleep disorders were found to be linked with many major clinical consequences [70,71,72]. It has become a wide psycho-social and public health issue globally which is linked with various diseases [73]. Thus, many researchers are producing different tools and approaches related to chronobiology and pharmacology to modulate the period, phase and amplitude of circadian rhythms to improve human health. The discovery of the self-sustained TTFLs has led to a new paradigm in our understanding of how organisms adapt to daily environmental cues [74].

### 6.1. Influence of the External Factors on Circadian Homeostasis and Diseases

**Figure 4:**
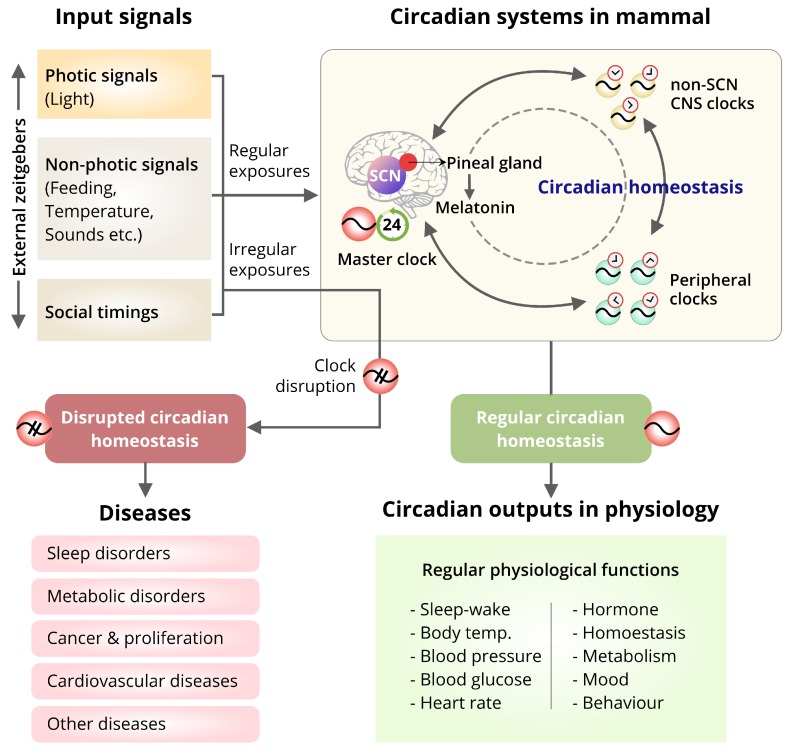
The association of the input signals, circadian homeostasis, and diseases. Intertwining interactions between the circadian clock and diseases are complex, and essentially depend not only on physiological factors alone, but also the influence of the different zeitgebers like, lights, sounds, temperature, feeding behaviors, lifestyles, and social timing received by the individual. Every individual clock needs to be synchronized within the tissue to sustain the stable phase-relationship to render the clock-mediated rhythmic information. The cellular clocks perceive the input signals by responding to the stimuli (input signals), integrate the phases to sense the temporal information and transmit the rhythmic information to other cells (circadian outputs) to adjust the indigenous clock timing with the external cues. This homeostatic mechanism drives the temporal regulations of different physiological functions. Disruption of such homeostasis leads to abnormal physiological activities and develops different diseases.

Circadian clocks are continuously interacting with external factors. The homeostasis of circadian clocks is crucial for persistence of many physiological activities, including sleep/wake cycles, feeding behavior, and body temperature [75,76]. Changes in our regular activities and feeding habits may play a part in disrupting the homeostasis of circadian rhythms and impair the associated physiological outputs (Figure 4). Disturbance of homeostasis has been reported to be associated with several physiological impairments and disease progression [42,57,77,78,79,80,81].

Precise synchronization of the circadian rhythmic systems to the 24-h a day necessitates regular adjustments driven by exposures to the various environmental time cues or zeitgebers [82,83]. Mammalian systems exhibit a typical “free-running period (FRP)” mode slightly different than 24-h [76,84,85,86]. The FRP expresses the SCN-based endogenous circadian rhythms without any corrective adjustments influenced by external cues. The interaction between circadian rhythms and its homeostasis on sleep/wake cycles were explained in the opponent process model of the sleep regulation theories [87]. This is a process in which sleep tendency increases with the duration of prior wake and is known as sleep homeostatic drive [87]. It begins to be accumulated immediately upon awakening and as the day progresses. These accumulated sleep drives are not manifested as explicit sleepiness. During daytime, the circadian rhythmic systems generate an alerting signal that counteracts the expression of sleep drive. In addition, at the end of the day, typically 1 to 2 h before the habitual bedtime, the circadian alerting signals alleviates. Thus, the balance between the two opponent processes shifts, and a person is used to feeling sleepy [87]. With the onset of sleep, those accumulated homeostatic sleep drives start dispersing. A healthy person exposed to a typical entrainment in accordance with regular, conventional schedule, the sleep homeostatic drives and the circadian rhythmic systems are synchronized with each other and with the 24-h solar day/night cycle [87].

In the mammalian circadian systems, a hormone called melatonin plays a crucial role in regulating the homeostasis and response to the light/dark cycles. Melatonin has the potential to modulate the timing of circadian rhythms and its outputs. In addition, it also plays an important role in synchronizing those circadian rhythmic outputs in accordance to the prevailing light/dark cycles. Melatonin is primarily synthesized in the pineal gland and enters to the blood stream in a rhythmic manner. The rhythmic synthesis of melatonin is driven by the SCN. Light exposure intervenes in the circadian signals to block the activities of the pineal gland [88,89,90,91]. High levels of melatonin can be found during night-time and low levels during the daytime [88,89,90,91]. Its secretion proportionally depends on the length of the darkness. Alterations in light/dark exposures or responsiveness to the seasonal changes cause a parallel change in the pattern of melatonin secretion. Those variations in melatonin secretion cause the changes of the body clocks [92,93,94]. The temporal regulation of melatonin secretion is closely associated with the timing of sleep propensity and decreases in core body temperature [95,96]. As the light/dark cycles have the greatest influence to the circadian rhythmic systems, the greatest phase delay is usually achieved when the light starts near the dim light melatonin onset (DLMO) and maximum phase advance is occurred after 7 to 8 h of light exposure after DLMO, near the core body temperature minimum [95,96]. The phase and amplitude of melatonin secretion are accounted as an arm of the biological clock [90]. The chronobiological properties of melatonin are primarily intended through SCN-based membrane G-protein-coupled MT1 and MT2 melatonin receptors [97]. The associations of various sleep-related actions presides in MT1 and MT2 receptors as well [93]. Melatonin is used to block the SCN-derived neuronal firing by acting on MT1 receptors [98]. Furthermore, the influence of melatonin on the sleep/wake cycles also shares similar mechanisms [99]. The melatonin-mediated phase-shifting effects are directed through SCN-based MT2 receptors [100]. Together, these two melatonin receptors were found to differentially modulate another SCN-based receptor: gamma-aminobutyric acid (GABA) receptors [101]. The GABA receptors were reported to influence both the phase shifts and the synchronizations of SCN clocks [102].

Frequent alterations to the exposure to the light/dark cycles facilitate the homeostatic functions of circadian rhythms to become out of synchronization with environmental timing. Furthermore, this leads to misalignments in circadian rhythmic behaviors [103,104,105,106,107,108]. The effects of circadian misalignment are compounded due to jet lag, frequent shift work, or any sort of irregular habitual behaviors such as abnormal feeding, excessive consumption of alcohol or caffeine-related interruptions to sleep that increases sleep debt to the bodies’ regular need [30,70,76,93,103,109,110,111,112].With the circadian rhythmic misalignment, the circadian alerting signals get reduced or even become absent during the daytime. Thus, during waking hours, homeostatic sleep drive may cause an anomaly in counteraction. This can also cause excessive sleepiness even when the individual has obtained a sufficient amount of sleep. The irregular habitual behaviors such as caffeine and alcohol consumption have the potential to modulate the circadian rhythms by disturbing the homeostasis. The period-lengthening effects are in response to chronic caffeine consumption habit [113,114,115] Caffeine was found to attenuate the regulation of adenylyl cyclase activity by extracellular adenosine. The activation of a G-protein subfamily, G*_i_α* subunit by adenosine was blocked by caffeine, and the production of cAMP were boosted [113,116,117]. As dynamic cAMP signaling is usually required for regular circadian timekeeping, and chronic modulation of cAMP turnover lengthens the period of cellular circadian rhythms [113,118], acute caffeine was found to reduce the rate of cAMP degradation, and facilitates in increasing calcium release from intracellular stores [113]. Similarly, alcohol consumption interacts with circadian rhythms also. Alcohol consumption is found to suppress melatonin production, thus causing reduced level of melatonin in the blood stream [119]. Low levels of melatonin have been associated with several disorders, majorly sleep disorders [89,120]. Therefore, different activities in opposition to our body’s endogenous rhythmic systems by disrupting the homeostatic drives can lead to significant circadian misalignments (Figure 4). It also causes various negative physiological consequences.

### 6.2. Effect of the Misalignment of the SCN-Master Clock and Peripheral Clocks on Diseases

The rhythmic oscillations are present not only in the SCN-master clock but also throughout the body. The SCN-driven output produces temporal signals broadcast widely in the different brain regions and peripheral tissues (as discussed in Section 4). Cumulatively, they exert broad effects at every level of organization, from genome regulation to control of protein synthesis, cell signaling and many other physiological functions [75]. Thus, misalignments of the synchronizations between these SCN-master clock and different tissue-specific peripheral clocks are tightly linked in diseases development across the system (Figure 4) [75,121].

#### 6.2.1. Sleep Disorders

The circadian rhythms have been recognized as a vital signature to drive the sleep/wake cycles primarily [122,123]. Sleep has also several prominent functions in our physiology, such as maintenance of metabolic homeostasis [124], clearance of neurotoxic waste byproducts [124], and DNA repairing [125]. Sleep alterations are associated with the transcriptional changes of core circadian regulators and their DNA-binding occupancy [126,127]. The core clock components, *Bmal1*, *Clock*, *Npas2*, *Per1*, *Per2*, *Cry1*, and *Cry2* have been linked to regulation of sleep onset and transition of wakefulness [128,129,130]. Targeted deletion and/or knocking-down the core clock components have been demonstrated in various alterations in sleep phenotypes including frequent increases in sleep fragmentation, sleep deprivation, and rapid switching between non-rapid eye movement and rapid eye movement sleep behavior [131]. A mutated residue (P385R) at highly conserved region was found to be associated with short sleep phenotype in human circadian transcriptional repressor DEC2 [28,132]. Clock disruptions and rhythmic desynchronizations among SCN-master clock, extra-SCN brain clocks, and peripheral clocks are largely associated with the poor sleep health and linked to neurodegenerative diseases and mood disorders [133]. The circadian rhythmic disruptions in mammals were also designated as a contributing factor in the aging process [60,61]. Disrupted core clock genes have been associated with the signature hallmark of early aging in mice [61]. Sleep and circadian rhythmic outputs are very tightly linked connecting to others physiological functions. Another distinct aspect of SCN-neuronal couplings is to rendering the rhythmic pacemakers highly stable to any stimuli causing shifts in peripheral tissue-clocks [9].

#### 6.2.2. Metabolic Diseases

Circadian rhythms have been well studied to control several metabolic processes, including oxidative phosphorylation [134], redox homeostasis [135], and lipid metabolism [136]. In several clinical studies, circadian misalignment was introduced to human subjects in a controlled laboratory setting and severe dysregulation of glucose homeostasis, insulin secretion, and appetitive control were observed [137,138]. The liver clock is highly entrainable with feeding behaviors and presence of light. In mouse models, obesity was induced by misaligning the feeding time and the indigenous clock time [139,140]. In this case, the indigenous body clock was desynchronized with the external cues. In another experiment with rodents, the alignment of the feeding time and activity through dietary restrictions at night helps them to protect from fatty liver buildup [141]. Also, similar strategies have appeared useful in improving human metabolic health [142]. A genome-wide association study indicated that the circadian gene ablation in the pancreas leads to *β*-cell failure and diabetes mellitus [143]. Genetic studies showed the liver clock disruptions results in fasting-induced hypoglycemia triggered by the impaired oxidative metabolism [144]. In many cases, sleep loss itself has been implicated in metabolic and proliferative diseases, although the effects are quite hard to characterize separately from circadian disruption as the two processes are highly interlinked [75]. Among the metabolic syndromes, energy uptake and expenditure, and neuronal activation and inhibition become imbalanced [145]. Shift workers face sleep deprivation that usually reduces the production of growth hormone and melatonin, reduces insulin production, and elevates cortisol levels [146]. Ultimately, these lead to obesity, diabetes, and then several cardio-respiratory diseases. Even in experiments with clocks, mutant mice exhibit the foundation of circadian rhythm-disrupted metabolic syndromes and consequences [143]. Metabolic disorders, eating disorders, and obesity are often associated with mood disorders in humans as well [147]. Individuals suffering from mood disorders receive benefit from strict daily routines and sleep quality maintenance scheduling [55,148]. These alignments of daily routines may help in adjusting and re-synchronizing the clocks in the body to preserve the integrity of the circadian systems and physiological homeostasis [149].

#### 6.2.3. Cancer

Clock disruptions have been marked as an exacerbating factor in tumorigenesis. Physiologically, disruption of circadian rhythms has been reported to contribute in lung tumorigenesis [81]. This is accelerated by multiple ways, including other hormonal effects, feeding behavior, metabolic alterations, as well as desynchronization of the circadian clock mechanisms. Such desynchronizations were mediated by the misalignment of the body’s indigenous clocks by external cues, which causes subsequent misalignments to other peripheral clocks residing in different tissues. In mice, whole-animal clock disruption of *Per2*, and *Bmal1* confirmed the role of circadian rhythmic disruption that promotes lung tumorigenesis in cooperation with *Kras* and *p53* [81]. Such homeostatic disruption caused significant activation of the expression of *cMyc*, *p53* and *Per2*. Studies also suggested that genetically disrupted circadian clocks with impaired melatonin synthesis and secretion were also found to be associated with tumor development [150]. Clock disruption caused suppression of the expression of the MT1 melatonin and glucocorticoid receptors in the liver that perturbs the liver peripheral clock by suppressing the hormone receptors [150]. The temporal expressions of *Per1*, *Bmal1* and *Dbp* were phase-shifted, and the expression of *Per2* was significantly up-regulated in liver [150]. An emerging epidemiologic and experimental evidence has implicated the link of desynchronized clock mechanisms with the cancer progression [62]. Studies with *Per2* mutant mice with severe dysregulation in cell cycle also revealed increased associations of radiation-induced lymphoma [62]. In contrast, disruption of the *Cry* genes in mice was implicated in tumor protection as it increased the predisposition to the cell death [151]. Again, DNA damage caused to shift the circadian oscillations by sequestrating the *Cry1*, mediated by deubiquitinase Herpes virus-associated ubiquitin specific protease [151]. Tumor cell glycolysis process is facilitated by the interaction among the core clock TFs and the oncogene *Myc*. Thus, the circadian disruption may be perceived as a sustained stressor associated with different diseases and their progressions.

#### 6.2.4. Cardiovascular Diseases

Almost all cardiovascular variables exhibit a day/night pattern [152]. Disruption of their rhythmic behaviors caused different cardiovascular diseases. The circadian rhythmic behaviors of the heart rate and vascular blood pressure were reported to get disrupted in mice with knockout of *Bmal1* and mutations in *Clock, Npas2* [153]. The deletion of *Bmal1* caused both the disruption of circadian clock and affects in disrupted cardiovascular rhythms. Desynchronization between the external cues and disruption of clock genes upon mutations modulate the stress related symptoms through sympathoadrenal path [153]. At molecular level, clock-mediated differential regulations were found for catecholamines and corticosteroids, driving their circadian variation [152,153]. This study strongly suggests that the clock may influence the time-dependent incidence of cardiovascular events by controlling the integration of selective asynchronous stress responses with an underlying circadian rhythm in cardiovascular function.

As the circadian clock disruptions and the diseases are tightly linked in many ways, it is quite important to understand the rhythmic processes and their anomalies deeply. However, the holistic identification and characterization of the all-inclusive molecular regulators, their networks, interplays at different layers and detailed mechanisms underpinning the circadian rhythmic oscillations and their tissue-specific synchronization are still not comprehensively understood [3,55,154]. Targeting the deep root is limited with prevailing interventions. There is no better existing approach to explain the entire dynamic landscape underlying those molecular regulators from the cellular perspective to the systems level [155,156]. The existing approaches are mostly lacking to consider the fine resolutions at the regulatory level and dynamic profiling at transcriptional, post-transcriptional, and then translational, and post-translational level. Many practices are merely based on knowledge despite lacking a strong scientific support. However, with the continuous efforts and advancements must help the scientists to deploy the thorough understanding of circadian rhythms into clinical practices including but not limited to the emergence of chronotherapeutics [20,74].

## 7. Recent Status of the Chronotherapy: A Potential Therapeutic Intervention

Chronotherapy is a way to treat illness according to the body’s indigenous timekeeping mechanisms of circadian clock [157]. For many years, chronotherapeutic managements for circadian rhythm-associated physiological disorders have been attempted. However, they have not emerged largely due to lack of scientific evidence. The emergence of chronotherapy aims to restore the misalignments of the SCN-master clock and the peripheral clocks with the external cues to adjust the circadian homeostasis [70,74,84]. It involves a variety of strategies that control exposure to environmental stimuli that influence the circadian homeostasis at systems level, mostly with non-pharmacological interventions [158].

To align the desynchronized SCN-clock and peripheral clocks, a behavioral approach (non-pharmacological chronotherapy) is used in which the circadian clock is reset by progressively delaying sleep/wake times by a few hours, until the desired sleep and wake times are achieved and maintained by adhering to a set sleep/wake schedule and good sleep hygiene practices [159]. One of the most commonly used non-pharmacological chronotherapy is the timed bright light exposure (BLE) to treat delayed sleeping phase syndrome [160]. BLE can reset the human circadian systems and can be predicted by the human phase-response curve. BLE in the early morning can advance the phase of the circadian rhythms, while BLE in the evening is capable of delaying the phase. In 1990, Rosenthal and colleagues showed that the study subjects had earlier sleep times and reported improvement in morning alertness after two weeks of daily morning (7:00 AM–9:00 AM) BLE at 2500 lux combined with restricted evening lights [161]. Even though the BLE is widely accepted as being effective, it is limited by the scientifically established standardized guidelines indicating the optimum exposure duration based on different clinical situations, intensity of the lights to be used and the timing of the exposure. The most commonly recommended approach is a broad-spectrum bright light of 2000–10,000 lux early morning (6:00 AM–8:00 AM) for approximately 1–3 h [162].

Such non-pharmacological chronotherapy demonstrated high degree of compliance and almost no side effects [163,164]. The behavioral approach of chronotherapeutic interventions are being used to treat mood disorders and sleep disorders by changing circadian rhythms of the patients, it is known as psychiatric chronotherapeutics [165,166]. To accomplish this intervention, external timekeeper or “zeitgeber” is needed. Based on certain evidence that light can indirectly prompt hormonal regulation, especially the distribution of the pineal hormone melatonin, through a non-visual photic input, ranging from special ganglion cells in the retina, ipRGCs to the SCN [37], light can be optimized therapeutically to reverse the disrupted circadian rhythm to treat sleep disorders especially. Light suppresses the segregation of melatonin, and thus the changes in melatonin level drive the sleep/wake rhythm in mammals [55]. Regrettably, there is no strong and straightforward evidence supporting their mechanisms. When night or darkness last longer, the length of the nocturnal melatonin segregation extends as well. Bright light in the morning advances the melatonin segregation whereas bright light in the evening delays it [55]. That is why patients treated with morning bright light get tired earlier in the evening. In patients with sleep disorders, the internal and external zeitgebers are not balanced, which leads to a shorter sleep duration and a lower sleep quality [111,158,167,168,169]. Chronotherapeutics such as BLE [158,167], wake therapy, and sleep phase advance derive benefit from the dependency of the hormonal rhythms on external zeitgebers and use artificial external ones to rebalance internal and external rhythms. Wake therapy is frequently reported as the fastest antidepressant known, and several studies proved that this antidepressive effect appears within hours [158,167].

Chronotherapeutics have a strong positive impact on reversing sleep disturbances which are one of the most frequent epiphenomena in major depression [158,167] and are associated with many good terms and healthy outcome. However, there is substantial evidence suggesting sleeping behavior, sleep architecture, and depressive symptoms significantly differ among children, adolescents, and adults, even sometimes across geography and races.

However, there is not enough scientific evidence beyond the relational effects of chronotherapeutics in various stages of lifespan, different geographical locations, or ethnic races. In future, the wide range of studies encompassing the scopes of the chronotherapy must be evolved. Also, chronotherapy will become an emerging therapeutic intervention for treating numerous clinical situations related to disturbances of the circadian rhythms.

## 8. Conclusions and Perspectives

In this review, we have highlighted the fundamental foundations, recent status, progressive advancements, and the potential futuristic indications to better understand the mammalian circadian clockwork, from molecules to systems. Certainly, circadian rhythms are quite complicated and systematic involving thousands of genes and multimodal regulatory layers. Eventually, understanding those deep-layered mechanisms must offer a plausible clue towards understanding the timely coordination of the different clocks. Even though it is tricky, intense, and challenging to establish a strong pathway backing for this synchronization and spatio-temporal dynamics among different clockworks within a living system, the more systematic integrity of different layers of molecular information is required to be studied altogether to understand the complex clockwork comprehensively. Thus, transcriptional, post-transcriptional, translational, post-translational, as well as wide-spectrum histone modifications, and protein–protein interactions must be studied all-inclusively to decode the deep molecular interplay among different layers of regulators for the circadian rhythm dynamics. Also, the detailed mechanistic insights for orchestration of the molecular oscillations from SCN, extra-SCN brain oscillators, and different distal peripheral tissues are yet to be vigorously studied. In addition, tissue-specific entrainments from different internal and external cues also need to be deeply investigated before making a concrete grip to modulate circadian rhythms for therapeutic purposes. Certainly, these deep insights and emerging quantitative strategies must help us to devise innovative therapeutic interventions and more precise, evidence-driven chronotherapeutics to plausibly manipulate the circadian rhythms at the dynamic scale from molecule to systems, thus to treat several disorders associated with circadian disturbances including one of the most neglected public health concerns, sleep disorders.

## References

[1] Granados-Fuentes D., Herzog E.D. (2013). The clock shop: Coupled circadian oscillators. Exp. Neurol..

[2] Partch C.L., Green C.B., Takahashi J.S. (2014). Molecular architecture of the mammalian circadian clock. Trends Cell Biol..

[3] Mure L.S., Le H.D., Benegiamo G., Chang M.W., Rios L., Jillani N., Ngotho M., Kariuki T., Dkhissi-Benyahya O., Cooper H.M. (2018). Diurnal transcriptome atlas of a primate across major neural and peripheral tissues. Science.

[4] Koike N., Yoo S.H., Huang H.C., Kumar V., Lee C., Kim T.K., Takahashi J.S. (2012). Transcriptional architecture and chromatin landscape of the core circadian clock in mammals. Science.

[5] Rey G., Cesbron F., Rougemont J., Reinke H., Brunner M., Naef F. (2011). Genome-wide and phase-specific DNA-binding rhythms of BMAL1 control circadian output functions in mouse liver. PLoS Biol..

[6] Schmutz I., Ripperger J.A., Baeriswyl-Aebischer S., Albrecht U. (2010). The mammalian clock component PERIOD2 coordinates circadian output by interaction with nuclear receptors. Genes Dev..

[7] Lamia K.A., Papp S.J., Ruth T.Y., Barish G.D., Uhlenhaut N.H., Jonker J.W., Downes M., Evans R.M. (2011). Cryptochromes mediate rhythmic repression of the glucocorticoid receptor. Nature.

[8] Padmanabhan K., Robles M.S., Westerling T., Weitz C.J. (2012). Feedback regulation of transcriptional termination by the mammalian circadian clock PERIOD complex. Science.

[9] Liu A.C., Welsh D.K., Ko C.H., Tran H.G., Zhang E.E., Priest A.A., Buhr E.D., Singer O., Meeker K., Verma I.M. (2007). Intercellular coupling confers robustness against mutations in the SCN circadian clock network. Cell.

[10] Cho H., Zhao X., Hatori M., Ruth T.Y., Barish G.D., Lam M.T., Chong L.W., DiTacchio L., Atkins A.R., Glass C.K. (2012). Regulation of circadian behaviour and metabolism by REV-ERB-*α* and REV-ERB-*β*. Nature.

[11] Sato T.K., Panda S., Miraglia L.J., Reyes T.M., Rudic R.D., McNamara P., Naik K.A., FitzGerald G.A., Kay S.A., Hogenesch J.B. (2004). A functional genomics strategy reveals Rora as a component of the mammalian circadian clock. Neuron.

[12] Preitner N., Damiola F., Zakany J., Duboule D., Albrecht U., Schibler U. (2002). The orphan nuclear receptor REV-ERB*α* controls circadian transcription within the positive limb of the mammalian circadian oscillator. Cell.

[13] Damiola F., Schibler U. (2003). Orphan nuclear receptors, molecular clockwork, and the entrainment of peripheral oscillators. Mol. Clocks Light Signal..

[14] Menet J.S., Rodriguez J., Abruzzi K.C., Rosbash M. (2012). Nascent-Seq reveals novel features of mouse circadian transcriptional regulation. elife.

[15] Suter D.M., Molina N., Gatfield D., Schneider K., Schibler U., Naef F. (2011). Mammalian genes are transcribed with widely different bursting kinetics. Science.

[16] Suter D.M., Molina N., Naef F., Schibler U. (2011). Origins and consequences of transcriptional discontinuity. Curr. Opin. Cell Biol..

[17] Terajima H., Yoshitane H., Ozaki H., Suzuki Y., Shimba S., Kuroda S., Iwasaki W., Fukada Y. (2017). ADARB1 catalyzes circadian A-to-I editing and regulates RNA rhythm. Nat. Genet..

[18] Ralph M.R., Menaker M. (1988). A mutation of the circadian system in golden hamsters. Science.

[19] Lowrey P.L., Shimomura K., Antoch M.P., Yamazaki S., Zemenides P.D., Ralph M.R., Menaker M., Takahashi J.S. (2000). Positional syntenic cloning and functional characterization of the mammalian circadian mutation tau. Science.

[20] Toh K.L., Jones C.R., He Y., Eide E.J., Hinz W.A., Virshup D.M., Ptáček L.J., Fu Y.H. (2001). An hPer2 phosphorylation site mutation in familial advanced sleep phase syndrome. Science.

[21] Gallego M., Virshup D.M. (2007). Post-translational modifications regulate the ticking of the circadian clock. Nat. Rev. Mol. Cell Biol..

[22] Hirota T., Lewis W.G., Liu A.C., Lee J.W., Schultz P.G., Kay S.A. (2008). A chemical biology approach reveals period shortening of the mammalian circadian clock by specific inhibition of GSK-3*β*. Proc. Natl. Acad. Sci. USA.

[23] Siepka S.M., Yoo S.H., Park J., Song W., Kumar V., Hu Y., Lee C., Takahashi J.S. (2007). Circadian mutant Overtime reveals F-box protein FBXL3 regulation of cryptochrome and period gene expression. Cell.

[24] Godinho S.I., Maywood E.S., Shaw L., Tucci V., Barnard A.R., Busino L., Pagano M., Kendall R., Quwailid M.M., Romero M.R. (2007). The after-hours mutant reveals a role for Fbxl3 in determining mammalian circadian period. Science.

[25] Belden W.J., Dunlap J.C. (2008). SIRT1 is a circadian deacetylase for core clock components. Cell.

[26] Cardone L., Hirayama J., Giordano F., Tamaru T., Palvimo J.J., Sassone-Corsi P. (2005). Circadian clock control by SUMOylation of BMAL1. Science.

[27] Xu Y., Padiath Q.S., Shapiro R.E., Jones C.R., Wu S.C., Saigoh N., Saigoh K., Ptáček L.J., Fu Y.H. (2005). Functional consequences of a CKI*δ* mutation causing familial advanced sleep phase syndrome. Nature.

[28] He Y., Jones C.R., Fujiki N., Xu Y., Guo B., Holder J.L., Rossner M.J., Nishino S., Fu Y.H. (2009). The transcriptional repressor DEC2 regulates sleep length in mammals. Science.

[29] Ebisawa T., Uchiyama M., Kajimura N., Mishima K., Kamei Y., Katoh M., Watanabe T., Sekimoto M., Shibui K., Kim K. (2001). Association of structural polymorphisms in the human period3 gene with delayed sleep phase syndrome. EMBO Rep..

[30] Partonen T. (2012). Clock gene variants in mood and anxiety disorders. J. Neural Transm..

[31] Albrecht U. (2017). Molecular mechanisms in mood regulation involving the circadian clock. Front. Neurol..

[32] De Leersnyder H., Claustrat B., Munnich A., Verloes A. (2006). Circadian rhythm disorder in a rare disease: Smith–Magenis syndrome. Mol. Cell. Endocrinol..

[33] Shi S.Q., Bichell T.J., Ihrie R.A., Johnson C.H. (2015). Ube3a imprinting impairs circadian robustness in Angelman syndrome models. Curr. Biol..

[34] Tsuchiya Y., Minami Y., Umemura Y., Watanabe H., Ono D., Nakamura W., Takahashi T., Honma S., Kondoh G., Matsuishi T. (2015). Disruption of Me CP 2 attenuates circadian rhythm in CRISPR/Cas9-based Rett syndrome model mouse. Genes Cells.

[35] Field M.D., Maywood E.S., O’Brien J.A., Weaver D.R., Reppert S.M., Hastings M.H. (2000). Analysis of clock proteins in mouse SCN demonstrates phylogenetic divergence of the circadian clockwork and resetting mechanisms. Neuron.

[36] Honma S. (2018). The mammalian circadian system: A hierarchical multi-oscillator structure for generating circadian rhythm. J. Physiol. Sci..

[37] Abbott K.S., Queener H.M., Ostrin L.A. (2018). The ipRGC-driven pupil response with light exposure, refractive error, and sleep. Optom. Vis. Sci..

[38] Hatori M., Gronfier C., Van Gelder R.N., Bernstein P.S., Carreras J., Panda S., Marks F., Sliney D., Hunt C.E., Hirota T. (2017). Global rise of potential health hazards caused by blue light-induced circadian disruption in modern aging societies. npj Aging Mech. Dis..

[39] Chew K.S., Renna J.M., McNeill D.S., Fernandez D.C., Keenan W.T., Thomsen M.B., Ecker J.L., Loevinsohn G.S., VanDunk C., Vicarel D.C. (2017). A subset of ipRGCs regulates both maturation of the circadian clock and segregation of retinogeniculate projections in mice. Elife.

[40] Buijs F.N., León-Mercado L., Guzmán-Ruiz M., Guerrero-Vargas N.N., Romo-Nava F., Buijs R.M. (2016). The circadian system: A regulatory feedback network of periphery and brain. Physiology.

[41] Mohawk J.A., Green C.B., Takahashi J.S. (2012). Central and peripheral circadian clocks in mammals. Annu. Rev. Neurosci..

[42] Eckel-Mahan K.L., Patel V.R., Mohney R.P., Vignola K.S., Baldi P., Sassone-Corsi P. (2012). Coordination of the transcriptome and metabolome by the circadian clock. Proc. Natl. Acad. Sci. USA.

[43] Honma S., Ono D., Suzuki Y., Inagaki N., Yoshikawa T., Nakamura W., Honma K.I. (2012). Suprachiasmatic nucleus: Cellular clocks and networks. Progress in Brain Research.

[44] Balsalobre A., Damiola F., Schibler U. (1998). A serum shock induces circadian gene expression in mammalian tissue culture cells. Cell.

[45] Panda S. (2016). Circadian physiology of metabolism. Science.

[46] Castelo-Szekely V., Arpat A.B., Janich P., Gatfield D. (2017). Translational contributions to tissue specificity in rhythmic and constitutive gene expression. Genome Biol..

[47] Antle M.C., Silver R. (2005). Orchestrating time: Arrangements of the brain circadian clock. Trends Neurosci..

[48] El Cheikh Hussein L., Mollard P., Bonnefont X. (2019). Molecular and Cellular Networks in The Suprachiasmatic Nuclei. Int. J. Mol. Sci..

[49] Lehman M.N., Silver R., Gladstone W., Kahn R.M., Gibson M., Bittman E.L. (1987). Circadian rhythmicity restored by neural transplant. Immunocytochemical characterization of the graft and its integration with the host brain. J. Neurosci..

[50] Ecker J.L., Dumitrescu O.N., Wong K.Y., Alam N.M., Chen S.K., LeGates T., Renna J.M., Prusky G.T., Berson D.M., Hattar S. (2010). Melanopsin-expressing retinal ganglion-cell photoreceptors: Cellular diversity and role in pattern vision. Neuron.

[51] Golombek D.A., Rosenstein R.E. (2010). Physiology of circadian entrainment. Physiol. Rev..

[52] Antle M.C., Smith V.M., Sterniczuk R., Yamakawa G.R., Rakai B.D. (2009). Physiological responses of the circadian clock to acute light exposure at night. Rev. Endocr. Metab. Disord..

[53] Guilding C., Piggins H.D. (2007). Challenging the omnipotence of the suprachiasmatic timekeeper: Are circadian oscillators present throughout the mammalian brain?. Eur. J. Neurosci..

[54] Colwell C.S. (2011). Linking neural activity and molecular oscillations in the SCN. Nat. Rev. Neurosci..

[55] Albrecht U. (2012). Timing to perfection: The biology of central and peripheral circadian clocks. Neuron.

[56] Chang A.M., Aeschbach D., Duffy J.F., Czeisler C.A. (2015). Evening use of light-emitting eReaders negatively affects sleep, circadian timing, and next-morning alertness. Proc. Natl. Acad. Sci. USA.

[57] Roenneberg T., Allebrandt K.V., Merrow M., Vetter C. (2012). Social jetlag and obesity. Curr. Biol..

[58] Schernhammer E.S., Laden F., Speizer F.E., Willett W.C., Hunter D.J., Kawachi I., Colditz G.A. (2001). Rotating night shifts and risk of breast cancer in women participating in the nurses’ health study. J. Natl. Cancer Inst..

[59] Pan A., Schernhammer E.S., Sun Q., Hu F.B. (2011). Rotating night shift work and risk of type 2 diabetes: Two prospective cohort studies in women. PLoS Med..

[60] Nakamura T.J., Nakamura W., Yamazaki S., Kudo T., Cutler T., Colwell C.S., Block G.D. (2011). Age-related decline in circadian output. J. Neurosci..

[61] Kondratov R.V., Kondratova A.A., Gorbacheva V.Y., Vykhovanets O.V., Antoch M.P. (2006). Early aging and age-related pathologies in mice deficient in BMAL1, the core componentof the circadian clock. Genes Dev..

[62] Fu L., Pelicano H., Liu J., Huang P., Lee C.C. (2002). The circadian gene Period2 plays an important role in tumor suppression and DNA damage response in vivo. Cell.

[63] Panda S., Antoch M.P., Miller B.H., Su A.I., Schook A.B., Straume M., Schultz P.G., Kay S.A., Takahashi J.S., Hogenesch J.B. (2002). Coordinated transcription of key pathways in the mouse by the circadian clock. Cell.

[64] Sobel J.A., Krier I., Andersin T., Raghav S., Canella D., Gilardi F., Kalantzi A.S., Rey G., Weger B., Gachon F. (2017). Transcriptional regulatory logic of the diurnal cycle in the mouse liver. PLoS Biol..

[65] Storch K.F., Lipan O., Leykin I., Viswanathan N., Davis F.C., Wong W.H., Weitz C.J. (2002). Extensive and divergent circadian gene expression in liver and heart. Nature.

[66] Nguyen T.T., Mattick J.S., Yang Q., Orman M.A., Ierapetritou M.G., Berthiaume F., Androulakis I.P. (2014). Bioinformatics analysis of transcriptional regulation of circadian genes in rat liver. BMC Bioinform..

[67] Le Martelot G., Canella D., Symul L., Migliavacca E., Gilardi F., Liechti R., Martin O., Harshman K., Delorenzi M., Desvergne B. (2012). Genome-wide RNA polymerase II profiles and RNA accumulation reveal kinetics of transcription and associated epigenetic changes during diurnal cycles. PLoS Biol..

[68] Vollmers C., Schmitz R.J., Nathanson J., Yeo G., Ecker J.R., Panda S. (2012). Circadian oscillations of protein-coding and regulatory RNAs in a highly dynamic mammalian liver epigenome. Cell Metab..

[69] Yan B., Guan D., Wang C., Wang J., He B., Qin J., Boheler K.R., Lu A., Zhang G., Zhu H. (2017). An integrative method to decode regulatory logics in gene transcription. Nat. Commun..

[70] Zee P.C., Attarian H., Videnovic A. (2013). Circadian rhythm abnormalities. Contin. Lifelong Learn. Neurol..

[71] Jagannath A., Taylor L., Wakaf Z., Vasudevan S.R., Foster R.G. (2017). The genetics of circadian rhythms, sleep and health. Hum. Mol. Genet..

[72] Santamaria F., Esposito M., Montella S., Cantone E., Mollica C., De Stefano S., Mirra V., Carotenuto M. (2014). Sleep disordered breathing and airway disease in primary ciliary dyskinesia. Respirology.

[73] Sutton C.E., Finlay C.M., Raverdeau M., Early J.O., DeCourcey J., Zaslona Z., O’Neill L.A., Mills K.H., Curtis A.M. (2017). Loss of the molecular clock in myeloid cells exacerbates T cell-mediated CNS autoimmune disease. Nat. Commun..

[74] Baiardi S., Cirignotta F., Cicolin A., Garbazza C., D’Agostino A., Gambini O., Giordano A., Canevini M., Zambrelli E., Marconi A.M. (2016). Chronobiology, sleep-related risk factors and light therapy in perinatal depression: The “Life-ON” project. BMC Psychiatry.

[75] Bass J., Lazar M.A. (2016). Circadian time signatures of fitness and disease. Science.

[76] Schulz P., Steimer T. (2009). Neurobiology of circadian systems. CNS Drugs.

[77] Ma D., Liu T., Chang L., Rui C., Xiao Y., Li S., Hogenesch J.B., Chen Y.E., Lin J.D. (2015). The liver clock controls cholesterol homeostasis through Trib1 protein-mediated regulation of PCSK9/Low density lipoprotein receptor (LDLR) axis. J. Biol. Chem..

[78] Sato S., Solanas G., Peixoto F.O., Bee L., Symeonidi A., Schmidt M.S., Brenner C., Masri S., Benitah S.A., Sassone-Corsi P. (2017). Circadian reprogramming in the liver identifies metabolic pathways of aging. Cell.

[79] Musiek E.S., Holtzman D.M. (2016). Mechanisms linking circadian clocks, sleep, and neurodegeneration. Science.

[80] Kitazawa M. (2013). Circadian rhythms, metabolism, and insulin sensitivity: transcriptional networks in animal models. Curr. Diabetes Rep..

[81] Papagiannakopoulos T., Bauer M.R., Davidson S.M., Heimann M., Subbaraj L., Bhutkar A., Bartlebaugh J., Vander Heiden M.G., Jacks T. (2016). Circadian rhythm disruption promotes lung tumorigenesis. Cell Metab..

[82] Czeisler C.A., Duffy J.F., Shanahan T.L., Brown E.N., Mitchell J.F., Rimmer D.W., Ronda J.M., Silva E.J., Allan J.S., Emens J.S. (1999). Stability, precision, and near-24-h period of the human circadian pacemaker. Science.

[83] Sack R.L., Lewy A.J., Blood M.L., Keith L.D., Nakagawa H. (1992). Circadian rhythm abnormalities in totally blind people: Incidence and clinical significance. J. Clin. Endocrinol. Metab..

[84] Sack R.L., Auckley D., Auger R.R., Carskadon M.A., Wright K.P., Vitiello M.V., Zhdanova I.V. (2007). Circadian rhythm sleep disorders: Part II, advanced sleep phase disorder, delayed sleep phase disorder, free-running disorder, and irregular sleep/wake rhythm. Sleep.

[85] Quera Salva M.A., Hartley S., Léger D., Dauvilliers Y.A. (2017). Non-24-h sleep–wake rhythm disorder in the totally blind: Diagnosis and management. Front. Neurol..

[86] Morgenthaler T.I., Lee-Chiong T., Alessi C., Friedman L., Aurora R.N., Boehlecke B., Brown T., Chesson A.L., Kapur V., Maganti R. (2007). Practice parameters for the clinical evaluation and treatment of circadian rhythm sleep disorders. Sleep.

[87] Edgar D.M., Dement W.C., Fuller C.A. (1993). Effect of SCN lesions on sleep in squirrel monkeys: Evidence for opponent processes in sleep/wake regulation. J. Neurosci..

[88] Pang S., Brown G., Grota L., Chambers J., Rodman R. (1977). Determination of N-acetylserotonin and melatonin activities in the pineal gland, retina, Harderian gland, brain and serum of rats and chickens. Neuroendocrinology.

[89] Lewy A. (2007). Melatonin and human chronobiology. Cold Spring Harbor Symposia on Quantitative Biology.

[90] Lewy A.J., Emens J., Jackman A., Yuhas K. (2006). Circadian uses of melatonin in humans. Chronobiol. Int..

[91] Grof E., Grof P., Brown G.M., Arato M., Lane J. (1985). Investigations of melatonin secretion in man. Prog. Neuro-Psychopharmacol. Biol. Psychiatry.

[92] Bartness T., Goldman B. (1989). Mammalian pineal melatonin: A clock for all seasons. Experientia.

[93] Brown G.M., Pandi-Perumal S.R., Trakht I., Cardinali D.P. (2009). Melatonin and its relevance to jet lag. Travel Med. Infect. Dis..

[94] Claustrat B., Brun J., Chazot G. (2005). The basic physiology and pathophysiology of melatonin. Sleep Med. Rev..

[95] Khalsa S.B.S., Jewett M.E., Cajochen C., Czeisler C.A. (2003). A phase response curve to single bright light pulses in human subjects. J. Physiol..

[96] Thompson A., Batterham A., Jones H., Gregson W., Scott D., Atkinson G. (2013). The practicality and effectiveness of supplementary bright light for reducing jet-lag in elite female athletes. Int. J. Sport. Med..

[97] Dubocovich M.L., Markowska M. (2005). Functional MT 1 and MT 2 melatonin receptors in mammals. Endocrine.

[98] Liu C., Weaver D.R., Jin X., Shearman L.P., Pieschl R.L., Gribkoff V.K., Reppert S.M. (1997). Molecular dissection of two distinct actions of melatonin on the suprachiasmatic circadian clock. Neuron.

[99] Von Gall C., Stehle J.H., Weaver D.R. (2002). Mammalian melatonin receptors: Molecular biology and signal transduction. Cell Tissue Res..

[100] Hunt A.E., Al-Ghoul W.M., Gillette M.U., Dubocovich M.L. (2001). Activation of MT2 melatonin receptors in rat suprachiasmatic nucleus phase advances the circadian clock. Am. J. Physiol.-Cell Physiol..

[101] Wan Q., Man H.Y., Liu F., Braunton J., Niznik H.B., Pang S.F., Brown G.M., Wang Y.T. (1999). Differential modulation of GABA A receptor function by Mel 1a and Mel 1b receptors. Nat. Neurosci..

[102] Liu C., Reppert S.M. (2000). GABA synchronizes clock cells within the suprachiasmatic circadian clock. Neuron.

[103] Arendt J. (2009). Managing jet lag: Some of the problems and possible new solutions. Sleep Med. Rev..

[104] Arendt J., Skene D.J. (2005). Melatonin as a chronobiotic. Sleep Med. Rev..

[105] Burgess H.J., Sharkey K.M., Eastman C.I. (2002). Bright light, dark and melatonin can promote circadian adaptation in night shift workers. Sleep Med. Rev..

[106] Gordon C.J., Comas M., Postnova S., Miller C.B., Roy D., Bartlett D.J., Grunstein R.R. (2018). The effect of consecutive transmeridian flights on alertness, sleep–wake cycles and sleepiness: A case study. Chronobiol. Int..

[107] Shiota M., Sudou M., Ohshima M. (1996). Using outdoor exercise to decrease jet lag in airline crewmembers. Aviat. Space Environ. Med..

[108] Fowler P.M., Duffield R., Morrow I., Roach G., Vaile J. (2015). Effects of sleep hygiene and artificial bright light interventions on recovery from simulated international air travel. Eur. J. Appl. Physiol..

[109] Sack R.L. (2009). The pathophysiology of jet lag. Travel Med. Infect. Dis..

[110] Daan S., Beersma D., Borbély A.A. (1984). Timing of human sleep: Recovery process gated by a circadian pacemaker. Am. J. Physiol.-Regul. Integr. Comp. Physiol..

[111] Wong W.S., Fielding R. (2011). Prevalence of insomnia among Chinese adults in Hong Kong: A population-based study. J. Sleep Res..

[112] Holst S.C., Valomon A., Landolt H.P. (2016). Sleep pharmacogenetics: Personalized sleep/wake therapy. Annu. Rev. Pharmacol. Toxicol..

[113] Burke T.M., Markwald R.R., McHill A.W., Chinoy E.D., Snider J.A., Bessman S.C., Jung C.M., O’Neill J.S., Wright K.P. (2015). Effects of caffeine on the human circadian clock in vivo and in vitro. Sci. Transl. Med..

[114] Oike H., Kobori M., Suzuki T., Ishida N. (2011). Caffeine lengthens circadian rhythms in mice. Biochem. Biophys. Res. Commun..

[115] Van Diepen H.C., Lucassen E.A., Yasenkov R., Groenen I., Ijzerman A.P., Meijer J.H., Deboer T. (2014). Caffeine increases light responsiveness of the mouse circadian pacemaker. Eur. J. Neurosci..

[116] Chen G., Van Den Pol A.N. (1997). Adenosine modulation of calcium currents and presynaptic inhibition of GABA release in suprachiasmatic and arcuate nucleus neurons. J. Neurophysiol..

[117] Conlay L.A., Conant J.A., Debros F., Wurtman R. (1997). Caffeine alters plasma adenosine levels. Nature.

[118] O’Neill J.S., Maywood E.S., Chesham J.E., Takahashi J.S., Hastings M.H. (2008). cAMP-dependent signaling as a core component of the mammalian circadian pacemaker. Science.

[119] Huang M.C., Ho C.W., Chen C.H., Liu S.C., Chen C.C., Leu S.J. (2010). Reduced expression of circadian clock genes in male alcoholic patients. Alcohol. Clin. Exp. Res..

[120] Karasek M., Winczyk K. (2006). Melatonin in humans. J. Physiol. Pharmacol..

[121] Turek F.W., Joshu C., Kohsaka A., Lin E., Ivanova G., McDearmon E., Laposky A., Losee-Olson S., Easton A., Jensen D.R. (2005). Obesity and metabolic syndrome in circadian Clock mutant mice. Science.

[122] Saper C.B., Scammell T.E., Lu J. (2005). Hypothalamic regulation of sleep and circadian rhythms. Nature.

[123] Fuller P.M., Gooley J.J., Saper C.B. (2006). Neurobiology of the sleep/wake cycle: Sleep architecture, circadian regulation, and regulatory feedback. J. Biol. Rhythm..

[124] Xie L., Kang H., Xu Q., Chen M.J., Liao Y., Thiyagarajan M., O’Donnell J., Christensen D.J., Nicholson C., Iliff J.J. (2013). Sleep drives metabolite clearance from the adult brain. Science.

[125] Zada D., Bronshtein I., Lerer-Goldshtein T., Garini Y., Appelbaum L. (2019). Sleep increases chromosome dynamics to enable reduction of accumulating DNA damage in single neurons. Nat. Commun..

[126] Laposky A.D., Bass J., Kohsaka A., Turek F.W. (2008). Sleep and circadian rhythms: Key components in the regulation of energy metabolism. FEBS Lett..

[127] Wisor J.P., Pasumarthi R.K., Gerashchenko D., Thompson C.L., Pathak S., Sancar A., Franken P., Lein E.S., Kilduff T.S. (2008). Sleep deprivation effects on circadian clock gene expression in the cerebral cortex parallel electroencephalographic differences among mouse strains. J. Neurosci..

[128] Borbély A.A. (1982). A two process model of sleep regulation. Hum. Neurobiol..

[129] Franken P., Dijk D.J. (2009). Circadian clock genes and sleep homeostasis. Eur. J. Neurosci..

[130] Franken P. (2013). A role for clock genes in sleep homeostasis. Curr. Opin. Neurobiol..

[131] Shaw P.J., Tononi G., Greenspan R.J., Robinson D.F. (2002). Stress response genes protect against lethal effects of sleep deprivation in Drosophila. Nature.

[132] Aran A., Einen M., Lin L., Plazzi G., Nishino S., Mignot E. (2010). Clinical and therapeutic aspects of childhood narcolepsy-cataplexy: A retrospective study of 51 children. Sleep.

[133] Peter-Derex L., Yammine P., Bastuji H., Croisile B. (2015). Sleep and Alzheimer’s disease. Sleep Med. Rev..

[134] Peek C.B., Affinati A.H., Ramsey K.M., Kuo H.Y., Yu W., Sena L.A., Ilkayeva O., Marcheva B., Kobayashi Y., Omura C. (2013). Circadian clock NAD+ cycle drives mitochondrial oxidative metabolism in mice. Science.

[135] Pekovic-Vaughan V., Gibbs J., Yoshitane H., Yang N., Pathiranage D., Guo B., Sagami A., Taguchi K., Bechtold D., Loudon A. (2014). The circadian clock regulates rhythmic activation of the NRF2/glutathione-mediated antioxidant defense pathway to modulate pulmonary fibrosis. Genes Dev..

[136] Grimaldi B., Bellet M.M., Katada S., Astarita G., Hirayama J., Amin R.H., Granneman J.G., Piomelli D., Leff T., Sassone-Corsi P. (2010). PER2 controls lipid metabolism by direct regulation of PPAR*γ*. Cell Metab..

[137] Buxton O.M., Cain S.W., O’Connor S.P., Porter J.H., Duffy J.F., Wang W., Czeisler C.A., Shea S.A. (2012). Adverse metabolic consequences in humans of prolonged sleep restriction combined with circadian disruption. Sci. Transl. Med..

[138] McHill A.W., Melanson E.L., Higgins J., Connick E., Moehlman T.M., Stothard E.R., Wright K.P. (2014). Impact of circadian misalignment on energy metabolism during simulated nightshift work. Proc. Natl. Acad. Sci. USA.

[139] Arble D.M., Bass J., Laposky A.D., Vitaterna M.H., Turek F.W. (2009). Circadian timing of food intake contributes to weight gain. Obesity.

[140] Kohsaka A., Laposky A.D., Ramsey K.M., Estrada C., Joshu C., Kobayashi Y., Turek F.W., Bass J. (2007). High-fat diet disrupts behavioral and molecular circadian rhythms in mice. Cell Metab..

[141] Hatori M., Vollmers C., Zarrinpar A., DiTacchio L., Bushong E.A., Gill S., Leblanc M., Chaix A., Joens M., Fitzpatrick J.A. (2012). Time-restricted feeding without reducing caloric intake prevents metabolic diseases in mice fed a high-fat diet. Cell Metab..

[142] Jakubowicz D., Froy O., Wainstein J., Boaz M. (2012). Meal timing and composition influence ghrelin levels, appetite scores and weight loss maintenance in overweight and obese adults. Steroids.

[143] Marcheva B., Ramsey K.M., Buhr E.D., Kobayashi Y., Su H., Ko C.H., Ivanova G., Omura C., Mo S., Vitaterna M.H. (2010). Disruption of the clock components CLOCK and BMAL1 leads to hypoinsulinaemia and diabetes. Nature.

[144] Perelis M., Marcheva B., Ramsey K.M., Schipma M.J., Hutchison A.L., Taguchi A., Peek C.B., Hong H., Huang W., Omura C. (2015). Pancreatic *β* cell enhancers regulate rhythmic transcription of genes controlling insulin secretion. Science.

[145] Gallou-Kabani C., Vigé A., Junien C. (2007). Lifelong circadian and epigenetic drifts in metabolic syndrome. Epigenetics.

[146] Spiegel K., Tasali E., Leproult R., Van Cauter E. (2009). Effects of poor and short sleep on glucose metabolism and obesity risk. Nat. Rev. Endocrinol..

[147] McIntyre R.S. (2009). Managing weight gain in patients with severe mental illness. J. Clin. Psychiatry.

[148] Frank E., Swartz H.A., Kupfer D.J. (2000). Interpersonal and social rhythm therapy: Managing the chaos of bipolar disorder. Biol. Psychiatry.

[149] Hlastala S.A., Frank E. (2006). Adapting interpersonal and social rhythm therapy to the developmental needs of adolescents with bipolar disorder. Dev. Psychopathol..

[150] Iwamoto A., Kawai M., Furuse M., Yasuo S. (2014). Effects of chronic jet lag on the central and peripheral circadian clocks in CBA/N mice. Chronobiol. Int..

[151] Ozturk N., Lee J.H., Gaddameedhi S., Sancar A. (2009). Loss of cryptochrome reduces cancer risk in p53 mutant mice. Proc. Natl. Acad. Sci. USA.

[152] Thosar S.S., Butler M.P., Shea S.A. (2018). Role of the circadian system in cardiovascular disease. J. Clin. Investig..

[153] Curtis A.M., Cheng Y., Kapoor S., Reilly D., Price T.S., FitzGerald G.A. (2007). Circadian variation of blood pressure and the vascular response to asynchronous stress. Proc. Natl. Acad. Sci. USA.

[154] Reinke H., Asher G. (2019). Crosstalk between metabolism and circadian clocks. Nat. Rev. Mol. Cell Biol..

[155] Yamada Y., Forger D. (2010). Multiscale complexity in the mammalian circadian clock. Curr. Opin. Genet. Dev..

[156] Susaki E.A., Ukai H., Ueda H.R. (2017). Next-generation mammalian genetics toward organism-level systems biology. NPJ Syst. Biol. Appl..

[157] Ballesta A., Innominato P.F., Dallmann R., Rand D.A., Levi F.A. (2017). Systems chronotherapeutics. Pharmacol. Rev..

[158] Lieverse R., Van Someren E.J., Nielen M.M., Uitdehaag B.M., Smit J.H., Hoogendijk W.J. (2011). Bright Light Treatment in Elderly patients with nonseasonal Major Depressive Disorder. Arch. Gen. Psychiatry.

[159] Weitzman E.D., Czeisler C.A., Coleman R.M., Spielman A.J., Zimmerman J.C., Dement W., Pollak C.P. (1981). Delayed sleep phase syndrome: A chronobiological disorder with sleep-onset insomnia. Arch. Gen. Psychiatry.

[160] Barion A., Zee P.C. (2007). A clinical approach to circadian rhythm sleep disorders. Sleep Med..

[161] Rosenthal N.E., Joseph-Vanderpool J.R., Levendosky A.A., Johnston S.H., Allen R., Kelly K.A., Souetre E., Schultz P.M., Starz K.E. (1990). Phase-shifting effects of bright morning light as treatment for delayed sleep phase syndrome. Sleep.

[162] Chesson A.L., Wise M., Davila D., Johnson S., Littner M., Anderson W.M., Hartse K., Rafecas J. (1999). Practice parameters for the treatment of restless legs syndrome and periodic limb movement disorder. Sleep.

[163] Levi F., Zidani R., Misset J.L. (1997). Randomised multicentre trial of chronotherapy with oxaliplatin, fluorouracil, and folinic acid in metastatic colorectal cancer. Lancet.

[164] Lévi F., Schibler U. (2007). Circadian rhythms: Mechanisms and therapeutic implications. Annu. Rev. Pharmacol. Toxicol..

[165] Kirschbaum I., Straub J., Gest S., Holtmann M., Legenbauer T. (2018). Short-term effects of wake-and bright light therapy on sleep in depressed youth. Chronobiol. Int..

[166] Suzuki M., Dallaspezia S., Locatelli C., Uchiyama M., Colombo C., Benedetti F. (2018). Does early response predict subsequent remission in bipolar depression treated with repeated sleep deprivation combined with light therapy and lithium?. J. Affect. Disord..

[167] Bais B., Kamperman A.M., van der Zwaag M.D., Dieleman G.C., van der Vliet H.W.H., Bijma H.H., Lieverse R., Hoogendijk W.J., Lambregtse-van den Berg M.P. (2016). Bright light therapy in pregnant women with major depressive disorder: Study protocol for a randomized, double-blind, controlled clinical trial. BMC Psychiatry.

[168] Calhoun S.L., Fernandez-Mendoza J., Vgontzas A.N., Liao D., Bixler E.O. (2014). Prevalence of insomnia symptoms in a general population sample of young children and preadolescents: Gender effects. Sleep Med..

[169] Stranges S., Tigbe W., Gómez-Olivé F.X., Thorogood M., Kandala N.B. (2012). Sleep problems: An emerging global epidemic? Findings from the INDEPTH WHO-SAGE study among more than 40,000 older adults from 8 countries across Africa and Asia. Sleep.

